# Epidemiology and resistance phenotypes of carbapenemase-producing *Klebsiella pneumoniae* in Greece, 2014 to 2016

**DOI:** 10.2807/1560-7917.ES.2018.23.31.1700775

**Published:** 2018-08-02

**Authors:** Irene Galani, Ilias Karaiskos, Irene Karantani, Vassiliki Papoutsaki, Sofia Maraki, Vassiliki Papaioannou, Polyzo Kazila, Helen Tsorlini, Nikoletta Charalampaki, Marina Toutouza, Helen Vagiakou, Konstantinos Pappas, Anna Kyratsa, Konstantina Kontopoulou, Olga Legga, Efthymia Petinaki, Helen Papadogeorgaki, Efrosini Chinou, Maria Souli, Helen Giamarellou, on behalf of the study collaborators

**Affiliations:** 1 Infectious Diseases Laboratory, 4th Department of Internal Medicine, National and Kapodistrian University of Athens, School of Medicine, Athens, Greece; 2 6th Department of Internal Medicine, Hygeia General Hospital, Athens, Greece; 3 Microbiology Laboratory, Hygeia General Hospital, Athens, Greece; 4 Department of Clinical Bacteriology, Parasitology, Zoonoses and Geographical Medicine, University Hospital of Heraklion, Heraklion, Greece; 5 Microbiology Department, KAT Hospital, Athens, Greece; 6 Cancer Hospital of Thessaloniki ‘THEAGENEIO’, Thessaloniki, Greece; 7 Microbiological Laboratories, Bacteriology Department ‘G. Papanikolaou’ General Hospital of Thessaloniki, Thessaloniki, Greece; 8 Department of Clinical Μicrobiology, Thriassio General Hospital, Elefsina, Athens, Greece; 9 Department of Microbiology, Hippokration Athens General Hospital, Athens, Greece; 10 Microbiology Laboratory, General Hospital of Athens ‘G. Gennimatas’, Athens, Greece; 11 Athens Naval Hospital, Athens, Greece; 12 Microbiology Laboratory, General Hospital of Corfu, Corfu, Greece; 13 Department of Microbiology, General Hospital of Thessaloniki ‘G. Gennimatas’, Thessaloniki, Greece; 14 Department of Microbiology, General Hospital of Lamia, Lamia, Greece; 15 Department of Microbiology, University Hospital of Larissa, Larissa, Greece; 16 Department of Microbiology, St Savvas Cancer Hospital, Athens, Greece; 17 The study collaborators are acknowledged at the end of the article

**Keywords:** carbapenem resistance, *Klebsiella pneumoniae*, carbapenemases, KPC, NDM, colistin, tigecycline, temocillin, ceftazidime/avibactam

## Abstract

A multicentre nationwide surveillance study was conducted in Greek hospitals to evaluate epidemiology of carbapenemase-producing *Klebsiella pneumoniae* clinical isolates, and their susceptibilities to last-line antibiotics. **Methods:** Minimum inhibitory concentrations (MICs) were evaluated by Etest, colistin MICs were also evaluated by broth microdilution SensiTest (now known as ComASP) Colistin. Carbapenemase genes were detected by PCR. Clonal relatedness was assessed by PFGE. Isolates were prospectively collected between November 2014 and April 2016, from 15 hospitals. **Results**: Among 394 isolates, *K. pneumoniae* carbepenemase (KPC) remained the most prevalent carbapenemase (66.5%). NDM was the second most prevalent (13.7%), identified in 12 hospitals, followed by VIM (8.6%). OXA-48- and double carbapenemase-producers remained rare (3.6%, 6.3%, respectively). Carbapenemase-producing *K. pneumoniae* isolates showed high resistance to last-line antibiotics. Gentamicin and colistin were the most active in vitro with 61.9% and 59.6% of the isolates to be inhibited at ≤ 2mg/L, followed by fosfomycin (susceptibility (S): 58.4%) and tigecycline (S: 51.5%). Ceftazidime/avibactam inhibited 99.6% of KPC and 100% of OXA-48-like-producing isolates, while temocillin was active against 58% of KPC isolates at urinary breakpoint of ≤ 32mg/L* and only 2.7% at systemic breakpoint of ≤ 8mg/L. NDM-producing isolates belonged mainly to one clone, whereas KPC, VIM, OXA-48 and double carbapenemase-producers were mainly polyclonal. **Conclusions**: KPC remains the predominant carbapenemase among *K. pneumoniae* in Greece, followed by NDM, whereas changing trends of resistance rates to last-line antimicrobials against carbapenemase-producing *K. pneumoniae* with the exception of ceftazidime/avibactam mandates continuing surveillance to support clinical practice.

## Introduction

Hospital infections caused by carbapenem-non-susceptible *Klebsiella pneumoniae* constitute a worldwide problem associated with high rates of treatment failure and mortality. In Greece, carbapenem-resistance in *K. pneumoniae* emerged in 2002 due to VIM and later, due to *K. pneumoniae* carbapenemase (KPC) carbapenemase production; both have become endemic [1,2]. The emergence of NDM-producing strains was reported in 2011 in the University Hospital of Ioannina (Epirus, Central Greece) [3]. Since then, sporadic cases have been observed in hospitals in Athens (Attica), Patras (Peloponnese, western Greece) and Larissa (Thessaly, central Greece), all belonging to multilocus sequence type (ST) 11 type [4-6]. The first OXA-48-like carbapenemase outbreak was detected in 2012 [7] and apart from single cases [8,9], no major epidemics have been recorded in Greece.

According to the most recent annual surveillance report from the European Centre for Disease Prevention and Control (ECDC) [10], Greece had the highest percentage of carbapenem-resistant isolates among invasive *K. pneumoniae* in Europe but with a decreasing trend from 68.2% in 2011 to 61.9% in 2015.

Treating carbapenem-non-susceptible *K. pneumoniae* infections is a major clinical challenge, because of the dearth of alternative drugs, often limited by poor bactericidal activity and/or high toxicity. Aminoglycosides, tigecycline, and the two revived antimicrobials, colistin and fosfomycin, are among the remaining options for clinicians to battle these difficult-to-treat infections [11]. Temocillin, a 6-alpha-methoxy derivative of ticarcillin, has also been suggested as a potential alternative treatment option for mild to moderately severe urinary tract infections caused by KPC-producing Enterobacteriaceae [12]. These antimicrobials in various combinations with or without a carbapenem [11,13] as well as the recently approved combination of ceftazidime/avibactam are included in therapeutic algorithms guided by the isolate MIC, the site and severity of infection, and the specific carbapenemase-encoding gene(s) [14]. It is of the utmost importance to monitor the spread of carbapenamase-producing *K. pneunoniae* and the dissemination of various carbapenemases in Greek hospitals in order to organise strategies for infection control and the application of antibiotic stewardship.

The primary aim of this study was to evaluate the epidemiology of contemporary carbapenemase-producing *K. pneumoniae* isolated in Greek hospitals, and their susceptibilities to the antimicrobials administrated in clinical practice, i.e. meropenem, colistin, tigecycline, fosfomycin, gentamicin, temocillin and ceftazidime/avibactam. The secondary aim was to evaluate the performance of Etest and VITEK2 (both from bioMérieux, Marcy-l’Étoile, France) in comparison to the reference standard microdilution method for colistin susceptibility across these contemporary multidrug-resistant isolates.

## Materials and methods

### Setting

Greece is a country in southern Europe with a population of ca 11 million as of 2016. Athens is the nation's capital and largest city (ca 3 million residents), followed by Thessaloniki (800,000 residents). Participating hospitals included 15 public and private tertiary- and secondary-care hospitals: eight in the Athens metropolitan area, three in Thessaloniki (northern Greece), one in Crete, the largest and most populous of the Greek islands in southern Greece, two in central Greece and one in western Greece, representing 12% of Greek hospitals.

The study was organised by the Hellenic Society of Chemotherapy in collaboration with the Infectious Diseases Laboratories of Hygeia Hospital, Athens and of the 4th Department of Internal Medicine of National and Kapodistrian University of Athens, where the susceptibility testing and molecular studies were performed. A nationwide multicentre surveillance network was formed by the microbiology laboratories of participating hospitals, which were asked to provide all consecutive single-patient *K. pneumoniae* clinical strains, isolated in a 6-month period and exhibiting non-susceptibility to any carbapenem (imipenem, meropenem or ertapenem) for further testing. These isolates were re-submitted for susceptibility testing as well as phenotypic and molecular detection of carbapenemases at a central laboratory.

Data on the source and the date of isolation as well as the initial susceptibility results at the local laboratories were also provided. All isolates were transferred to the Microbiology Laboratory of Infectious Diseases of Hygeia Hospital and kept frozen at −80 °C until the day of testing.

### Phenotypic methods for detecting carbapenemase activity

The combination disk test was used for screening carbapenemase production using meropenem 10 μg disks (BIO-RAD, Marne La Coquotte, France) with or without inhibitors (phenyl boronic acid (PBA), ethylenediaminetetraacetic acid (EDTA) and cloxacillin) as recommended by the European Committee on Antimicrobial Susceptibility Testing (EUCAST) [15]. Temocillin 30μg disk (Liofilchem srl, Roseto degli Abruzzi, Italy) was applied as a phenotypic indicator of OXA-48 production, when a tentative inhibition zone diameter < 11mm was seen in the absence of synergy with PBA and EDTA. A meropenem disk with PBA and EDTA was also included to detect double carbapenemase producers (KPC and VIM), which have emerged in Greek hospitals since 2009 [16].

All isolates with negative combination disk test were further examined for carbapenemase production by the carbapenem inactivation method (CIM), a method that has 100% negative predictive value for carbapenemase production in Enterobacteriaceae [17]. To perform the CIM, a 10 μg meropenem disk was immersed in a suspension of the tested isolate and incubated for a minimum of 2 h at 35 °C. After incubation, the disk was placed on a Mueller-Hinton agar plate inoculated with the susceptible *Escherichia coli* ATCC 29522 strain. If the bacterial isolate produced a carbapenemase, the meropenem in the susceptibility disk was inactivated, allowing uninhibited growth of the susceptible *E. coli* strain after 16 h of incubation at 35 °C. Disks incubated in suspensions that did not contain any carbapenemase yielded a clear inhibition zone.

### Molecular analysis of carbapenemase genes

Genotypic confirmation of carbapenemase production was conducted in all studied isolates and consisted of simplex in-house PCR assays targeting *bla*_KPC_, *bla*_NDM_, *bla*_VIM_, *bla*_IMP_ and *bla*_OXA-48_ with specific primers and conditions (Supplement 1). Template DNA was extracted from bacteria grown in Luria Bertani broth for 18 h using NucleoSpin Tissue kit (Machery-Nagel GmbH, Duren, Germany).

### Susceptibility testing

Confirmation of the species and MIC determination of ampicillin/sulbactam, piperacillin/tazobactam, cefoxitin, ceftazidime, ceftriaxone, cefepime, aztreonam, imipenem, meropenen, amikacin, gentamicin, ciprofloxacin, levofloxacin, tigecycline, fosfomycin, colistin and trimethoprim/sulfamethoxazole was performed by VITEK2. Additionally, the MICs of meropenem, colistin, tigecycline, fosfomycin, gentamicin and temocillin were determined by Etest and ceftazidime/avibactam by Liofilchem MIC Test Strip (Liofilchem srl), according to the manufacturer’s instructions. During the course of this study, the joint Clinical and Laboratory Standards Institute (CLSI)-EUCAST Polymyxin Breakpoints Working Group recommended that the reference method for colistin MIC determination be the ISO-standard broth microdilution method (20776–1) [18]. Thus, we additionally evaluated colistin MICs by the commercially available broth microdilution SensiTest Colistin (now marketed as ComASP Colistin (Liofilchem srl) according to the manufacturer's instructions.

*E. coli* ATCC 25922 and *K. pneumoniae* ATCC 700603 were included in all experiments for quality control, and all results were within accepted ranges. Results were interpreted according to EUCAST recommendations [19]. All isolates were sub-cultured twice before testing.

Multidrug-resistant (MDR), extensively drug-resistant (XDR) and pandrug-resistant (PDR) strains were characterised as per criteria described by ECDC and the United States’ Centers for Disease Control and Prevention (CDC). For this we used susceptibility data of all the above-mentioned 18 antimicrobials [20].

### Comparison of methods used for colistin minimum inhibitory concentration determination

Obtained colistin MICs were compared with the results of Etest and VITEK2 to determine essential and categorical agreement and very major error (VME) and major error (ME) rates.

Essential agreement was defined as MIC result within plus or minus one two-fold dilution from the reference result. This was adjusted for differences in the range of MICs that could be determined by the respective systems. A VME was defined when isolates were categorised as susceptible by Etest or VITEK2 but resistant by the broth microdilution (used as the reference method). An ME was defined when isolates were resistant by Etest or VITEK2 but susceptible by the broth microdilution. Unacceptable levels were > 1.5% for VME and > 3% for ME, as recommended in the CLSI document M23-A4 [21].

### PCR-based screening for the plasmid-mediated colistin resistance *mcr*-1/*mcr*-2 genes

Screening the entire collection of colistin-non-susceptible *K. pneumoniae* isolates (n = 159) for plasmid-mediated colistin resistance genes, was performed by a duplex PCR protocol optimised at the Danish National Food Institute, using primers described previously from Liu et al. [22] and Xavier et al. [23] (Supplement 1).

### PFGE typing

Genetic relatedness among carbapenemase-producing *K. pneumoniae* isolates was evaluated by PFGE analysis of chromosomal restriction fragments obtained following cleavage with *Spe*I (New England BioLabs Inc., GmbH Frankfurt am Main, Germany). A dendrogram was generated from the homology matrix with a coefficient of 1.5% using the unweighted pair-group method with arithmetic mean (UPGMA) to describe the relationships among PFGE profiles. Isolates were considered to belong to the same PFGE group if their Dice similarity index was ≥ 80% [24].

## Results

**Figure 1:**
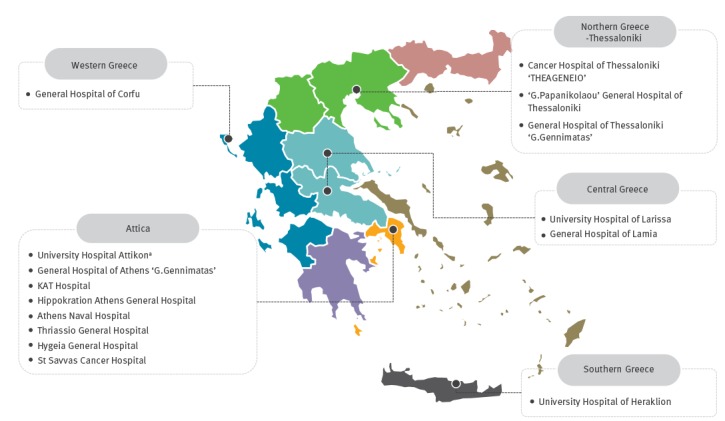
Geographical map showing the location of participating hospitals providing *Klebsiella pneumoniae* clinical isolates, Greece, 2014–2016 (n = 15)

^a^University General Hospital Attikon contributed 32 isolates from patients hospitalised in the 4th Department of Internal Medicine/Division of Infectious Diseases.

Between 1 November 2014 and 30 April 2016, 15 hospitals from six Greek cities contributed a median number of 20 consecutive isolates each to the study (Figure 1). During the study period, 394 *K. pneumoniae* isolates were collected (Table 1). They originated from different clinical specimens: urine (n = 168), blood (n = 86), lower respiratory tract secretions (n = 60), pus (n = 55), cerebrospinal fluid (n = 3) and unknown (n = 22).

**Table 1:** Carbapenem-non-susceptible *Klebsiella pneumoniae* clinical isolates submitted by participating hospitals, number of confirmed carbapenemase-producers and type of carbapenemase, by hospital, Greece, 2014­–2016 (n = 394)

Participating hospitals	Carbapenem-non-susceptible *K. pneumonia* isolates submitted	Confirmed carbapenemase-producing *K. pneumoniae* isolates										Other mechanism of carbapenem resistance	
		KPC		NDM		VIM		OXA-48-like		KPC + VIM or other dual combination			
	n	n	%	n	%	n	%	n	%	n	%	n	%
**Athens metropolitan area**	**167**	**103**	**61.7**	**25**	**15.0**	**20**	**12.0**	**12**	**7.2**	**7**	**4.2**	**0**	**0.0**
University General Hospital Attikon^a^	32	15	46.9	10	31.2	1	3.1	4	12.5	2	6.2	0	0.0
General Hospital of Athens ‘G. Gennimatas’	25	19	76.0	2	8.0	0	0.0	4	16.0	0	0.0	0	0.0
KAT Hospital	48	34	70.8	2	4.2	8	16.7	1	2.1	3^b^	6.2	0	0.0
Hippokration Athens General Hospital	20	13	65.0	4	20.0	2	10.0	0	0.0	1^c^	5.0	0	0.0
Athens Naval Hospital	18	12	66.7	3	16.7	0	0.0	3	16.7	0	0.0	0	0.0
Thriassio General Hospital	7	2	28.6	0	0.0	5	71.4	0	0.0	0	0.0	0	0.0
Hygeia General Hospital	14	6	42.8	3	21.4	4	28.6	0	0.0	1^c^	7.1	0	0.0
St Savvas Cancer Hospital	3	2	66.7	0	0.0	1	33.3	0	0.0	0	0.0	0	0.0
**Thessaloniki**	**90**	**60**	**66.7**	**7**	**7.8**	**5**	**5.6**	**2**	**2.2**	**13**	**14.4**	**3**	**33.3**
Cancer Hospital of Thessaloniki ‘THEAGENEIO’	43	30	69.8	1	2.3	4	9.3	0	0.0	5	11.6	3	7.0
‘G. Papanikolaou’ General Hospital of Thessaloniki	27	17	63.0	1	3.7	1	3.7	0	0.0	8	29.6	0	0.0
General Hospital of Thessaloniki ‘G. Gennimatas’	20	13	65.0	5	25.0	0	0.0	2	10.0	0	0.0	0	0.0
**Crete**	**77**												
University Hospital of Heraklion	77	65	84.4	5	6.5	6	7.8	0	0.0	1	1.3	0	0.0
**Central Greece**	**39**	**31**	**79.5**	**1**	**2.6**	**2**	**5.1**	**0**	**0.0**	**4**	**10.3**	**1**	**2.6**
University Hospital of Larissa	20	18	90.0	0	0.0	2	10.0	0	0.0	0	0.0	0	0.0
General Hospital of Lamia	19	13	68.4	1	5.3	0	0.0	0	0.0	4	21.1	1	5.3
**Western Greece**	**21**												
General Hospital of Corfu	21	3	14.3	17	81.0	0	0.0	0	0.0	0	0.0	1	4.8
**Total**	**394**	**262**	**66.5**	**54**	**13.7**	**34**	**8.6**	**14**	**3.6**	**25**	**6.3**	**5**	**1.3**

*K. pneumoniae*: *Klebsiella pneumoniae*; KPC: *Klebsiella pneumoniae* carbapenemase; NDM: New Delhi metallo-beta-lactamase; VIC: Verona integron-mediated metallo-beta-lactamase.

^a^ University Hospital Attikon contributed 32 isolates from patients hospitalised in the 4th Department of Internal Medicine/Division of Infectious Disease.

^b^ One isolate with KPC and OXA-48-like carbapenemase production.

^c^ These isolates were NDM and OXA-48-like carbapenemase producers.

The combination disk test detected 389 (98.7%) carbapenemase-producing isolates, with 263 (66.8%) exhibiting a class A, 90 (22.8%) a class B, 22 (5.6%) a class A and B carbapenemase phenotype and 14 (3.6%) an OXA-48-like phenotype. Five isolates (1.3%) exhibited a negative combination disk test result and were further examined by the CIM method, which was also negative and excluded the possibility of carbapenemase production.

Carbapenemase production was confirmed in all 389 isolates (98.7%) with a positive combination disk test, including 262 (66.5%), 54 (13.7%), 34 (8.6%) and 14 (3.6%) cases of *bla*_KPC_, *bla*_NDM_, *bla*_VIM_ and *bla*_OXA-48_-like genes, respectively. Additionally, 25 (6.3%) isolates harboured two carbapenemase genes, 22 (5.6%) both *bla*_KPC_ and *bla*_VIM_, two (0.5%) *bla*_NDM_ and *bla*_OXA-48_-like and one isolate (0.3%) *bla*_KPC_ and *bla*_OXA-48_-like. Isolates producing KPC or NDM along with OXA-48-like carbapenemases were falsely interpreted as only KPC- or NDM-producing isolates by the combination disk test. None of the carbapenemase genes examined in this study was detected in the five isolates (1.3%) with negative combination disk and CIM test result. Those carbapenemase-negative isolates exhibited meropenem/imipenem MICs of 0.5-4 mg/L and 0.25-0.5 mg/L respectively.

KPC enzymes, detected in 66.5% of isolates, were the most frequent carbapenemases in all but two hospitals, with a rate ranging between 14.3% and 90.0%. NDM carbapenemase was the second most frequent overall (13.7%) and was the most prominent only in one hospital (81.0%) in western Greece (Corfu General Hospital). In four hospitals (three in Athens area and one in Thessaloniki), NDM-producers were isolated at a rate of ≥ 20% while in three hospitals (two in the Athens area and one in central Greece) none of the isolates was an NDM-producer. VIM enzymes were detected in 8.6% of the *K. pneumoniae* isolates and were the most prominent class in one hospital of Athens area. OXA-48-like enzymes were detected in 14 isolates (3.6%) in five hospitals, four in the Athens area and one in Thessaloniki.

Susceptibilities of isolates interpreted according to EUCAST breakpoints are shown in Table 2. Nineteen (4.8%) isolates exhibited a PDR phenotype, 124 (31.5%) exhibited an XDR phenotype, and the remaining 251 (63.7%) an MDR phenotype. Gentamicin and colistin were the most active drugs in vitro, with 61.9% and 59.6% of the isolates found to be inhibited at ≤ 2mg/L, followed by fosfomycin (susceptibility (S): 58.4%) and tigecycline (S: 51.5%). Fosfomycin was the most active agent against NDM (S: 81.5%) and OXA-48 *K. pneumoniae* isolates (S: 78.6%), while gentamicin was the most active against KPC plus VIM- (S: 90.1%) and KPC-producers (S: 69.5%). Colistin was active against 77.8% of the NDM producers and was the most active drug against the VIM-producing isolates (S: 61.8%) (Table 2). Co-trimoxazole (SXT) was active against 22.1% of the *K. pneumoniae* isolates.

**Table 2:** Susceptibilities of carbapenem-non-susceptible *Klebsiella pneumoniae* isolates with regards to the mechanism of carbapenem resistance, Greece, 2014–2016 (n = 394)

Isolates (number)	Antimicrobial agent																						PDR	
	MEM			CST			TG			FOS			GM			CZA			TEM					
	%	MIC_50_	MIC_90_	%	MIC_50_	MIC_90_	%	MIC_50_	MIC_90_	%	MIC_50_	MIC_90_	%	MIC_50_	MIC_90_	%	MIC_50_	MIC_90_	% (≤ 32mg/L)^a^	% (≤ 8mg/L)^b^	MIC_50_	MIC_90_	n	%
**All (n = 394)**	2.8	> 32	> 32	59.6	1	> 16	51.5	1	4	58.4	32	256	61.9	2	> 256	ND	ND	ND	ND	ND	ND	ND	19	4.8
**KPC (n = 262)**	1.1	> 32	> 32	61.1	1	> 16	51.9	1	4	57.3	32	512	69.5	2	32	99.6	1	2	58.0	2. 7	32	64	12	4.6
**NDM (n = 54)**	5.6	> 32	> 32	77.8	1	> 16	66.7	1	2	81.5	16	256	42.6	128	> 256	ND	ND	ND	ND	ND	ND	ND	1	1.9
**VIM (n = 34)**	14.7	> 32	> 32	61.8	1	> 16	38.2	2	8	47.1	64	128	38.2	64	> 256	ND	ND	ND	ND	ND	ND	ND	3	8.8
**OXA-48 (n = 14)**	0.0	> 32	> 32	42.9	> 16	> 16	71.4	1	8	78.6	16	128	28.6	128	> 256	100.0	1	1	ND	ND	ND	ND	1	7.1
**KPC + VIM (n = 22)**	4.5	> 32	> 32	9.0	> 16	> 16	27.0	2	2	27.3	64	128	90.1	2	8	ND	ND	ND	ND	ND	ND	ND	1	4.5

CST: colistin; CZA: ceftazidime/avibactam; FOS: fosfomycin; GM: gentamicin; KPC: *Klebsiella pneumoniae* carbapenemase; MEM: meropenem; MIC: minimum inhibitory concentration; ND: not determined; NDM: New Delhi metallo-beta-lactamase; PDR: pandrug-resistant; TEM: temocillin; TG: tigecycline; VIM: Verona integron-mediated metallo-beta-lactamase.

^a^ Percentage of susceptibility at the British Society for Antimicrobial chemotherapy (BSAC) urinary breakpoint.

^b^ Percentage of susceptibility at the BSAC systemic breakpoint.

### Comparison of methods used for colistin minimum inhibitory concentration determination

**Figure 2:**
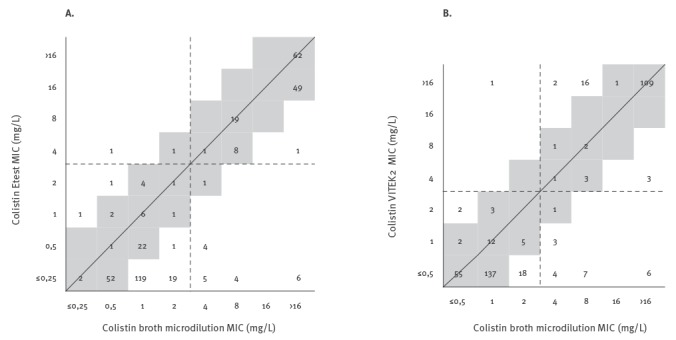
Scattergram comparing (A) Etest minimum inhibitory concentrations and (B) VITEK2 minimum inhibitory concentrations with broth microdilution minimum inhibitory concentrations for colistin, tested against the 394 contemporary *Klebsiella pneumoniae* clinical isolates, Greece, 2014–2016

Discrepancies were observed in colisin MIC values reported via the Etest vs the reference broth microdilution SensiTest Colistin (Figure 2). A trend towards lower colistin MICs was noted for Etest (Table 3), resulting in fewer colistin non-susceptible isolates (142 vs 159) and a categorical agreement of 94.7%. Although there was an acceptable rate (> 90%) for the categorical agreement, a high percentage of VME (19 false susceptibles, 4.8%) was observed, which was above the criterion of ≤ 1.5% proposed by CLSI [21]. Etest produced MICs that were 1, 2, 3, and > 3 log_2_ dilutions lower than those determined by SensiTest Colistin for 28.4%, 22.3%, 20.8% and 5.8% of the isolates, respectively, resulting in a low rate of essential agreement (58.4%).

MIC: minimum inhibitory concentration.

The dashed line represents the EUCAST breakpoint for susceptibility (≤ 2 mg/L). The diagonal line indicates absolute agreement. Shaded area indicates essential agreement. Very major errors are indicated in bold.

**Table 3:** Distribution of minimum inhibitory concentration values of carbapenem-non-susceptible *Klebsiella pneumoniae* isolates grouped by the mechanism of carbapenem resistance, Greece, 2014–2016 (n = 394)

*Klebsiella pneumoniae* resistance genotype (n)	Antibiotic	Number of isolates inhibited at (mg/L)												
		≤ 0.125	0.25	0.5	1	2	4	8	16	32	64	128	256	≥ 512
**All** **(n = 394)**	MEM	0	0	3	2	6	17	25	32	51	258^a^	NA	NA	NA
	CST (Etest)	106	95	29	11	9	12	19	50	48	7	1	2	5
	CST (SensiTest)	NA	3^b^	56	153	23	12	27	2	118^a^	NA	NA	NA	NA
	FOS	0	0	0	0	0	3	21	101	105	79	36	13	36
	TG	1	4	49	149	139	37	10	3	0	0	0	0	2
	GM	0	0	6	53	185	42	5	9	7	9	9	11	58
***bla*_KPC_** **(n = 262)**	MEM	0	0	0	0	3	11	14	21	34	179^a^	NA	NA	NA
	CST (Etest)	80	61	18	7	5	10	12	27	31	5	0	2	4
	CST (SensiTest)	NA	2^b^	38	106	14	8	18	1	75^a^	NA	NA	NA	NA
	FOS	0	0	0	0	0	1	11	59	79	56	20	9	27
	TG	0	3	35	98	96	24	4	2	0	0	0	0	0
	GM	0	0	5	46	132	40	2	8	4	5	0	1	19
	CZA	5	13	76	113	46	8	0	1	0	0	0	0	0
	TEM	0	0	0	0	0	0	7	32	113	96	13	1	0
***bla*_NDM_** **(n = 54)**	MEM	0	0	0	0	0	0	3	6	12	33^a^	NA	NA	NA
	CST (Etest)	22	16	4	1	1	1	2	3	4	0	0	0	0
	CST (SensiTest)	NA	0	8	29	5	2	3	0	7^a^	NA	NA	NA	NA
	FOS	0	0	0	0	0	0	7	24	13	0	3	2	5
	TG	1	0	4	31	13	4	1	0	0	0	0	0	0
	GM	0	0	0	1	22	0	0	1	0	2	5	6	17
***bla*_VIM_** **(n = 34)**	MEM	0	0	1	1	3	4	3	4	1	17^a^	NA	NA	NA
	CST (Etest)	5	10	5	2	1	1	4	2	2	1	0	0	1
	CST (SensiTest)	NA	1^b^	6	12	2	2	4	1	6^a^	NA	NA	NA	NA
	FOS	0	0	0	0	0	1	1	5	9	11	5	0	2
	TG	0	1	5	7	10	5	4	1	0	0	0	0	1
	GM	0	0	0	2	11	2	0	0	2	1	2	2	12
***bla*_OXA-48-like_** **(n = 14)**	MEM	0	0	0	0	0	0	3	1	3	7^a^	NA	NA	NA
	CST (Etest)	5	1	1	0	0	0	0	6	1	0	0	0	0
	CST (SensiTest)	NA	0	3	1	2	0	0	0	8^a^	NA	NA	NA	NA
	FOS	0	0	0	0	0	1	0	9	1	1	1	1	0
	TG	0	0	1	9	2	0	1	0	0	0	0	0	1
	GM	0	0	0	2	2	0	0	0	1	0	2	1	6
	CZA	0	0	6	7	1	0	0	0	0	0	0	0	0
***bla*_KPC_+*bla*_VIM_** **(n = 22)**	MEM	0	0	0	1	0	0	2	0	0	19^a^	NA	NA	NA
	CST (Etest)	0	2	0	0	0	0	1	10	8	0	1	0	0
	CST (SensiTest)	NA	0	1	1	0	0	1	0	19^a^	NA	NA	NA	NA
	FOS	0	0	0	0	0	0	1	4	1	8	6	1	1
	TG	0	0	1	5	14	2	0	0	0	0	0	0	0
	GM	0	0	0	1	18	0	3	0	0	0	0	0	0
***bla*_NDM_+*bla*_OXA-48-like_** **(n = 2)**	MEM	0	0	0	0	0	0	0	0	1	1^a^	NA	NA	NA
	CST (Etest)	1	1	0	0	0	0	0	0	0	0	0	0	0
	CST (SensiTest)	NA	0	0	2	0	0	0	0	0	NA	NA	NA	NA
	FOS	0	0	0	0	0	0	0	0	1	1	0	0	0
	TIG	0	0	0	0	2	0	0	0	0	0	0	0	0
	GM	0	0	0	0	0	0	0	0	0	1	0	0	1
***bla*_KPC _+ *bla*_OXA-48-like_** **(n = 1)**	MEM	0	0	0	0	0	0	0	0	0	1^a^	NA	NA	NA
	CST (Etest)	0	0	0	0	0	0	0	1	0	0	0	0	0
	CST (SensiTest)	NA	0	0	0	0	0	0	0	1^a^	NA	NA	NA	NA
	FOS	0	0	0	0	0	0	0	0	0	0	1	0	0
	TG	0	0	0	0	0	1	0	0	0	0	0	0	0
	GM	0	0	0	0	0	0	0	0	0	0	0	0	1
	CZA	0	0	0	0	1	0	0	0	0	0	0	0	0
**non-CP (n=5)**	MEM	0	0	2	0	0	2	0	0	0	1^a^	NA	NA	NA
	CST (Etest)	1	2	0	0	0	0	0	0	1	1	0	0	0
	CST (SensiTest)	NA	0	0	2	0	0	1	0	2^a^	NA	NA	NA	NA
	FOS	0	0	0	0	0	0	1	0	1	2	0	0	1
	TG	0	0	1	1	2	1	0	0	0	0	0	0	0
	GM	0	0	1	1	0	0	0	0	0	0	0	0	3
	CZA	1	1	1	2	0	0	0	0	0	0	0	0	0
	TEM	1	0	2	0	0	0	0	0	1	1	0	0	0

CST: colistin; CST (Etest): minimum inhibitory concentration (MIC) as determined by Etest; CST (SensiTest): MIC as determined by SensiTest Colistin; CZA: ceftazidime/avibactam; FOS: fosfomycin; GM: gentamicin; MEM: meropenem; NA: not available; TEM: temocillin; TG: tigecycline.

^a^ Upper limit of determination, corresponds to ≥.

^b^ Lower limit of determination, corresponds to ≤.

Dark grey shading represents susceptible isolates by European Committee on Antimicrobial Susceptibility Testing (EUCAST) 2018 breakpoint. Light grey shading represents intermediate category according EUCAST 2018 breakpoint.

For colistin MIC values reported via the VITEK2 system (Figure 2), the categorical and the essential agreement rates were 94.4% and 84.0% respectively; 45.2% of the isolates had MICs equal to those determined by broth microdilution, while 38.8% of the isolates had MICs that were 1 log_2_ dilution higher or lower. Although categorical agreement was acceptable (> 90%), high percentage of VME (21 false susceptible, 5.3%) was observed, which was above the criterion of ≤ 1.5%.

Temocillin MIC distributions for the 262 KPC-producing isolates tested are shown in Table 3. Overall, only seven of the KPC-producing isolates (2.7%) were susceptible to temocillin, according to the British Society for Antimicrobial Chemotherapy (BSAC) systemic breakpoint (MICs ≤ 8 mg/L). At the higher BSAC urinary breakpoint (≤ 32 mg/L), 152 (58.0%) isolates were susceptible to temocillin (Table 2). However, 110 isolates (42.0%) remained non-susceptible with MICs > 32 mg/L. On the contrary, ceftazidime-avibactam was very active against isolates carrying *bla*_KPC_, or *bla*_OXA-48-like_, or both (MIC_50_/_90_, 0.5/2 mg/L; Table 1). This combination inhibited 99.6% of the non-MBL isolates at the breakpoint of ≤ 8mg/L.

Meropenem retained activity against 2.8% of *K. pneumoniae* isolates (MICs ≤ 2mg/L), while 18.8% (n = 74) exhibited moderately elevated carbapenem MICs (4–16 mg/L). Discrepancies were observed in meropenem MIC values reported via the VITEK2 system vs the Etest. Forty-five isolates (11.4%), all carbapenemase producers, exhibiting MICs 0.5–8 mg/L by Etest were categorised as resistant (MIC > 8mg/L) to meropenem by VITEK2. Additionally, 31 isolates (7.9%) with an Etest MIC of 16 mg/L, were categorised as resistant with a MIC > 8mg/L by VITEK2. These strains were reported as non-susceptible to carbapenems by the microbiology laboratories applying VITEK2 [25].

### PFGE typing

PFGE genotyping revealed a prevalent PFGE profile among KPC-producing *K. pneumoniae*, designated A, detected in 49.2% of the isolates, consisting of several variants (A1 to A9), all of which were detected in more than one centre. A second PFGE profile, designated B, consisting of three variants (B1 to B3), included 15 isolates, while a third PFGE profile, designated C, was detected only in two centres in Thessaloniki area, and consisted of two variants (C1 and C2), including 12 and three isolates, respectively. Twenty-four more profiles each included 2–10 isolates.

The 34 VIM-, the 22 KPC- and VIM- and the 14 OXA-48-like – producing *K. pneumoniae* isolates were multiclonal, with 17, 3 and 7 clones respectively, differing between hospitals and different clones present within a single hospital, with no particular clone prevailing.

On the contrary, PFGE genotyping of NDM-producing isolates demonstrated great genetic similarity in the 52 (96.3%) isolates (dominant clone A), consisting of three main variants (A1 to A3), two of which were detected in more than one centre. Additionally, two isolates, each with distinct PFGE profile, were also detected (1.9%). Moreover, two isolates carrying both *bla*_NDM_ and *bla*_OXA-48-like_ belonged to dominant clone A.

## Discussion

This nationwide, multicentre study showed that among 394 contemporary carbapenem-non-susceptible consecutive *K. pneunoniae* isolates collected at 15 hospitals from six cities in Greece, KPC remains the most prevalent carbapenemase (66.5%), followed by NDM (13.7%). VIM has declined, from being the exclusive carbapenemase detected in Greece until 2006 [1], to 8.4% in 2016 and is followed by double carbapenemase production (5.6%) and OXA-48 (3.6%). These findings are in accordance with those from the European Survey on Carbapenemase-Producing Enterobacteriaceae (EuSCAPE) project, conducted between November 2013 and April 2014, in which Grundmann et al. reported for the first time that NDM was the second-ranking carbapenemase in Greece [26]. In a previous published nationwide surveillance study from January 2011 to June 2012, among 119 Greek hospitals, the prevalent mechanism of carbapenem resistance in *K. pneumoniae* isolates was KPC (82.6%), followed by VIM (9.7%), while the concurrent production of KPC and VIM was noted in 7.7% of the isolates [27].

The Greek situation differs greatly from other European countries such as Spain, France, Germany, Turkey, Romania and Belgium, where OXA-48, most often linked with community-onset healthcare associated sources, is the most frequently encountered carbapenemase [26,28-31].

In terms of molecular typing, we identified ≥ 80% similarity among 96.3% of the NDM isolates, indicative of a single clone, expanding within and between 12 hospitals from different regions in Greece. This is in accordance with previous reports that one ST11 *bla*_NDM_-positive clone has quickly and successfully established its presence in four other Greek hospitals [3,4,32].

In the nationwide surveillance reported by Maltezou et al., it was observed that colistin, gentamicin and tigecycline resistance among carbapenem-resistant *K .pneumoniae* was 23.0%, 19.7% and 22.4%, respectively [27]. In our collection of isolates, colistin non-susceptibility rate mounted to 40.4%, and is probably related to chromosomal mutations, as no *mcr-1*-positive isolates were identified among the non-susceptible strains tested. The referred mean colistin non-susceptibility rate is much higher compared to the 28.3% observed by the EuSCAPE project among 36 participating countries [26]. This increasing trend could be ascribed to increased consumption of colistin in Greece from 0.0024 defined daily doses (DDD) per 1,000 patient-days (in 2001) to 0.0480 (in 2008) and in 0.0949 (in 2015) according to data available from the European Surveillance of Antimicrobial Consumption Network (ESAC-NET) database [33]. On the other hand, the suboptimal methods for susceptibility testing used in previous studies, which underestimated the level of resistance, could be an additional explanation for the increased rate of colistin resistance in our study (method-dependent resistance). It is of great importance to emphasise that antimicrobial susceptibility testing of colistin has been fraught with difficulties, which resulted in the necessity for the recently issued recommendations from CLSI and EUCAST [15]. Although not initially included in the aim of our study, colistin susceptibility testing with the gold-standard method of broth microdilution was performed and evaluation of performance of Etest and VITEK2 was also conducted and concluded in high rates of VME across both methods; both Etest and VITEK2 underestimated colistin MIC values and understated resistance [15,34,35].

Antimicrobial susceptibility testing confirmed MICs to temocillin > 8mg/L in nearly all KPC-producing *K. pneumoniae* isolates, precluding its use for systemic infections. However, when applying the urinary tract infection breakpoint of ≤ 32 mg/L, 58.4% of KPC-producers were susceptible, suggesting a possible role of temocillin in the treatment of urinary tract infections due to those isolates. Conversely, a variable susceptibility profile was observed for fosfomycin, depending on enzyme type production and ranged from 27.3% to 81.5%. In a systemic review, the reported susceptibility of fosfomycin in *K. pneumomiae*, mainly including KPC-producing *K. pneumoniae* ranged between 39.2% and 100% [36]. Intravenous fosfomycin, a revived antibiotic, has been used in carbapenem resistant Gram-negative infections, but has been associated with rapid development of resistance in vitro, and therefore monotherapy should be avoided [11]. Consequently, carbepenem- and colistin-sparing regimens for infections with MDR and XDR pathogens are welcome in settings with high resistance rates to last-line antimicrobials.

The combination ceftazidime/avibactam was active against 99.6% of KPC/OXA-48 *K. pneumoniae* isolates. Only one KPC-producing isolate was non-susceptible (MIC 16mg/L), for which further characterisation is necessary to confirm the mechanism of resistance. Production of KPC represents the main mechanism of carbapenem resistance among *K. pneumoniae* in Greek hospitals and this carbapenemase appears to be very well inhibited by avibactam. Thus the recently introduced combination represents a valuable treatment option for infections caused by KPC-producing *K. pneumoniae* isolates, including those caused by organisms resistant to most other antimicrobial agents, but knowledge of the underlying mechanism of resistance is required for its use [14].

In our collection, 2.8% of the *K. pneumoniae* isolates were susceptible to meropenem (MIC ≤ 2 mg/L), 10.7% were intermediately resistant (MIC 4–8 mg/L), while another 8.1% exhibited an MIC of 16 mg/L. For these isolates (21.6%), carbapenems are likely to maintain their clinical efficacy, especially in combination regimens [25,37]. In countries with a high incidence of carbapenemase-producing Enterobacteriaceae, like Greece, it seems imperative for an accurate method for meropenem MIC determination to be performed besides automated methods, as MIC values are important predictors of carbapenem effectiveness. The major limitation of the present multicentre surveillance study concerns the limited number of the participating Greek hospitals (12%), mainly attributed to financial limitations. Nevertheless, public and private tertiary- and secondary-care hospitals located in all major Greek cities were purposely included in order to represent the current situation in Greece. On the other hand, advantages of the present study include the simultaneous evaluation of the in vitro activity of all clinically relevant antimicrobials, the comparison of performance of three different methods for colistin susceptibility testing, the number of contemporary clinical isolates upon which obtained results were based, and most importantly, the use of a central laboratory for the microbiological studies, so that results could be comparable.

To conclude, this study demonstrated that in nosocomial carbapenem-non-susceptible strains of *K. pneumoniae* consecutively collected from Greek hospitals: (i) KPC enzyme remained the predominant carbapenemase; (ii) NDM, surprisingly when compared with results from previous years, was the second-ranking carbapenemase; (iii) NDM-harbouring isolates belonged to a single clone, while the KPC, VIM, double carbapenemase producing and OXA-48 isolates were polyclonal; (iv) colistin MIC determination by broth microdilution is imperative and (v) the steeply increasing resistance to last-line antimicrobials such as colistin in Greece mandates the necessity of continuous surveillance as well as the application of strict contact precautions along with antimicrobial stewardship.

## *Erratum

Due to an error, ≤ 32mg/L was mistakenly reported as ≤ 2mg/L. This was corrected on 10 August 2018.
